# Qualitative study of mental health attribution, perceptions and care-seeking in Kampala, Uganda

**DOI:** 10.4102/sajpsychiatry.v28i0.1690

**Published:** 2022-05-31

**Authors:** John M. Bwanika, Charlotte Hawkins, Louis Kamulegeya, Patricia Onyutta, Davis Musinguzi, Audrey Kusasira, Elizabeth K. Musoke, Jascintha Kabeega

**Affiliations:** 1 Department of Research, The Medical Concierge Group, Kampala, Uganda; 2 Infectious Diseases Institute Limited, Kampala, Uganda; 3 Department of Anthropology, University College London, London, United Kingdom; 4 The Medical Concierge Group Limited, Kampala, Uganda; 5 Auckland University of Technology, Auckland, New Zealand; 6 CATChA Team, Kampala, Uganda; 7 Department of Psychiatry, Naguru Hospital, Kampala, Uganda

**Keywords:** mental health disorders, mental health services, care-seeking, mental health attribution, Uganda

## Abstract

**Background:**

Mental health problems contribute to a substantial proportion of the global burden of disease. In Uganda, the World Health Organization estimates that 2.2 million people are affected by mental health disorders. Further research is needed to highlight people’s views about mental health in order to ensure that services are appropriate, accessible and effective.

**Aim:**

This qualitative study aimed to explore perceptions, experiences and care-seeking preferences to inform stakeholders looking to provide contextually appropriate mental health programmes.

**Setting:**

A diverse neighbourhood in central Kampala, Uganda.

**Methods:**

The authors conducted 56 in-depth semi-structured interviews with people over the age of 37 years from November 2018 to May 2019.

**Results:**

Participants discussed interpersonal and systemic issues that affect mental health in their community and the existing coping mechanisms that people employ. Social factors were often associated with mental health problems, with 36% of participants attributing them to economic stressors in particular. Mental health services were often perceived to be unavailable, costly or stigmatised, which can mean that care-seeking is delayed until problems become severe. Some people said they prefer to turn to prayer (25%) or counselling within their family or community (12.5%).

**Conclusion:**

Mental health problems are often attributed to socioeconomic factors, which can also hinder access to services. An understanding of perceptions about mental health can help to align programmes for appropriateness and effectiveness. Our study suggests that beneficial additional services for people living in low-income urban settings in Uganda could include those which are free, community-based or offering financial support.

## Introduction

Mental, neurological and substance use disorders (MNS) contribute to a substantial proportion of the global burden of disease and have been found to account for 10.4% of ‘disability-adjusted life years’ (DALYs).^1^ In a 2018 report, the World Health Organization (WHO) observed an ‘escalating burden of mental disorders’ around the world.^2^ This report states that mental health is determined by multiple social, psychological and biological factors, with outcomes for both individuals and their communities.^2^

To date, there have been no countrywide epidemiological studies on the prevalence of mental health disorders in Uganda,^3,4^ and there is inadequate data on hospital utilisation.^5^ However, a recent systematic review has found that 24% of the population are affected by depression and anxiety, demonstrating they are common mental disorders affecting roughly 1 in 4 people^4^; the authors suggest that their study likely underestimates prevalence.^4^ Overall, the WHO estimates that there are 2.2 million people affected by mental health problems in Uganda, with only 9% of them able to access care.^5^ The WHO has also observed that most low-income countries spend the majority of mental health budgets on psychiatric hospitals, which serve only a small proportion of those who need care.^6^ In Uganda, government and private services are concentrated in the capital city, Kampala, with specialised mental health services distributed to regional referral hospitals.^7^ In contrast to the aim of the 2001 Abuja Declaration,^8^ in which African Union countries pledged to allocate 15% of their national budgets to the health sector, Uganda spends 6.9% of GDP on health, with 17% of that allocated to non-communicable diseases (NCDs).^5^ Less than 1% of the total health budget goes into mental healthcare, 55% of which is spent on the one National Psychiatric Hospital, Butabika.^5,9^ Despite efforts to enhance and decentralise mental health services, improve regional capacities and integrate mental health service delivery, awareness and accessibility of services within communities remains limited.^10^

The WHO Mental Health and Poverty Project (MHaPP) Research Programme Consortium have advised that further research is needed to highlight community views surrounding mental health problems in order to ensure that mental health services reach more people in Uganda.^10^ In line with this, our study explored the perceptions and care-seeking practices around mental health in Uganda in a selected Kampala community in order to inform stakeholders looking to implement mental health projects. The study focused on beliefs about what causes mental health disorders, the social consequences of those beliefs and their influence on help-seeking behaviour. Formative research such as our study has been shown to provide essential insights for health initiatives to direct funding and implementation approaches.^11^ An understanding of care-seeking preferences can inform service design and referrals.^12^ Typically, care-seeking is a ‘dynamic process’ determined by various sociocultural and demographic factors, as well as service accessibility.^12^ Pathways to care therefore differ according to context and the prevailing conceptualisations regarding mental health. For example, in Uganda, as in other African contexts, studies have emphasised the prevalence of initial consultation with traditional and religious healers, the barriers and delays this presents to formal healthcare and the need for collaboration between biomedical and traditional or religious mental health services.^13,14,15,16^ However, our study shows that traditional or religious explanations of mental health may overlap with medical treatment-seeking, depending on its perceived availability. The distinction between biomedical and traditional healing models is therefore not clear-cut, instead reflecting what has been usefully conceptualised by medical anthropologists as a ‘continuum’ of care,^17^ with alternative options sought ‘simultaneously and sequentially’ on a pragmatic basis.^17^

This interview study was conducted within a longer-term ethnography on ageing, health and phone use in Kampala, Uganda. This article is part of a collaboration between this PhD project and the Medical Concierge Group (TMCG), a leading digital health organisation in Uganda. The findings are presented in consideration of the development of contextually relevant mental health interventions in Uganda, such as social psychiatry and public health programmes.^18^

## Methods

This article is based on findings from 56 in-depth, semi-structured and informal interviews conducted between November 2018 and May 2019 with people over the age of 37 in a low-income area in Kampala, here given the pseudonym Lusozi to protect participants’ anonymity. This was part of a 16-month ethnography about people’s experiences of mid-life, particularly related to health and mobile phones. Ethnographic methods rely on longer term fieldwork within the participants’ setting.^19^ This methodology is flexible and allows for an inductive and holistic grounded approach^20^; being open-ended, this approach is uniquely suited to building a nuanced understanding of social aspects of mental health problems. For example, informal face-to-face interviews are an appropriate research method to gain insight into personal perceptions and experiences of research participants.^20^ This methodology is particularly well suited for highlighting illness concepts, experiences, contextual factors and care models, which can provide a crucial evidence base for mental health interventions.^21^ Our study thereby gained a broad perspective on how mental health is understood by research participants, people not necessarily pre-disposed to respond to research specifically on mental health as they were encountered in the neighbourhood setting. Our study focused on people over 37 as they may often act as advisors within their family and community and yet their perspective is often overlooked in youth-focused research. Within longer interviews, questions about mental health focused on general knowledge and experience of mental health problems: stress, depression and alcoholism; stigma; treatment options; counselling or advice.

### Setting

The study setting is a low-income neighbourhood in central Kampala. According to 2014 census figures, the area has a total population of around 32 000 people.^22^ Participants had lived in the area for an average of 17 years. Residents come from all over the country, and the region beyond; they often call the neighbourhood ‘the United States of Kampala’, reflecting the diversity of this densely populated urban setting. Local leadership estimates that half of the people living in Lusozi are from Northern Uganda, some of them having been displaced during the recent civil war in the region that ended in the late 2000s. Most interviews were conducted in participant’s homes, but those with health workers took place in health clinics or in the nearby government hospital.

### Sample

The interview study recruited participants based on a convenience sampling approach as part of a wider ethnographic study, in which the researchers were familiar with the community. The convenience sample ensured that the group of respondents were not necessarily predisposed to seeking research and information regarding mental health. Our study also included six health workers based in the area, one of whom is a psychiatrist at the local government hospital, two are Psychiatric Clinical Officers (PCOs) and three are health workers in private clinics (Table 1).

**TABLE 1:** Research sample population.

Interviewee categories	Number of participants (N = 56)	%	Community leaders	Psychiatric clinicians	Health workers in private clinics
Men (37–68)	16	28	4	-	-
Women (39–84)	33	60	1	-	-
Younger men (20–30) focus group	1	2	-	-	-
Health worker	6	11	-	3	3

### Data collection

Interviewees were asked questions about their knowledge and experience of mental health in general terms, as well as questions about their personal experiences of mental health problems or those of their family and community. Initial questions, which sought an understanding of mental health in general terms, would often initiate a response about mental health disorders such as schizophrenia,^23^ which manifest with ‘psychotic symptoms’^24^ including hallucination.^25^ This is why questions also specifically asked about ‘nonpsychotic symptoms’ of common mental health disorders such as anxiety and depression.^5^ The interviews lasted for 1 h on average. A total of 13 interviews were audio recorded and transcribed verbatim and 41 were reconstructed from detailed notes taken during the interview. Interviews were carried out by the author and supported by a co-investigator who grew up in the area. A total of 25 of the interviews were conducted in English and 31 were translated and back translated from Luo or Luganda to English by the co-investigator, depending on the preference of the research participant.

### Data analysis

Thematic data analysis was guided by a grounded inductive approach, identifying patterns in the data.^26^ To increase validity and reduce the limitations of a single researcher, four of the authors (C.H., J.M.B., P.O. and J.K.) reviewed and analysed available data to inform a discussion about the findings and reach a consensus about the coding themes. Thematic coding of the data on NVivo software was then reviewed and agreed by these four authors. Findings were then further sense checked by the co-author, J.K., who is the head of psychiatry at the government hospital.

### Ethical considerations

The ethnographic study was approved by Makerere School of Social Sciences (MAKSS) and the Ugandan National Council of Science & Technology (UNCST) in November 2017, reference number: 10.17.096. 29/01/2018. The researcher gained full consent from local leadership prior to conducting the study, in particular the Local Council 3 (LC3) Chairwoman for the area and the hospital director. All participants gave full informed consent to take part in the study. The names of participants are concealed, and the study setting is anonymised with a pseudonym in order to further disguise participants’ identities.

## Results

This first results section offers a brief summary of findings related to the causality of mental health problems, including associations with financial stress and ‘overthinking’, alcohol and drugs and supernatural causes such as ‘witchcraft’. The coding themes are outlined in Table 2. The article then focuses on reporting findings related to perceptions around treatment-seeking, including awareness of available hospital services and perceived limitations and proposed solutions for overcoming these limitations; the themes are shown in Table 3. Some participants (*n* = 7) explained how they would prefer to seek mental health advice from someone they know in their family or community than from a medical doctor. Many also advocated for religious solutions to mental health problems (*n* = 14), such as prayer and conversion to born-again Christianity. Issues related to social stigma towards people with mental health problems suggest that additional optional, confidential and accessible mental health services and information could be beneficial for people in Kampala, particularly those with limited incomes.

**TABLE 2:** Mental health attribution discussion themes.

Mental health attribution themes	Sub-themes
Alcohol and Drugs (‘njagga’)	Marijuana
	Peer Pressure
Economic issues	Unemployment
	Poverty
	School fees
	Gender
Stress / ‘Overthinking’	Overthinking
Supernatural causes	Ancestral punishment
	Family curses
	Co-wives
	Witchcraft
	Spiritual attacks
Family issues	Marital issues
Health conditions	HIV
	TB

HIV, human immunodeficiency virus; TB, tuberculosis.

**TABLE 3:** Care-seeking preferences discussion themes.

Care-seeking preferences	Sub-themes
Faith-based	Prayer
	Religious counsel
	Conversion to born-again Christianity
Medical treatment	Hospitals, for example, ‘Butabika’
	Service availability / limitations
Social	Family / community-based counselling
	Advice to ‘stop over-thinking’
Traditional healers	Urban / rural distinction

### Mental health attribution

The research population is outlined in Table 1. During interviews, mental health disorders with psychotic symptoms were sometimes attributed to family and marital issues (*n* = 4), diseases such as tuberculosis and human immunodeficiency virus (*n* = 6) or more commonly to spiritual issues (*n* = 11), whereas common mental health disorders were more likely to be associated with ‘thought disorders’ (*n* = 14) in response to social problems. Many participants (*n* = 20) attribute mental health problems to economic stressors. This includes issues such as underemployment, pressure to pay school fees and housing insecurity. Some participants explained how financial responsibilities can result in severe depression, as in the case of this 65-year-old grandmother being described by a local leader and counsellor for women:

‘[*S*]he lived to the extent of almost committing suicide, because she had nowhere to go, no one to help, nowhere! … she is depressed, totally depressed. You know, at least, even if you have nothing but your children, and you really have hope in your grandchild, but again the grandchild is not studying, there is no money … so that thing at times I think it can even cause mental illness.’ (Participant 1, 49 year old woman, Women’s Leader)

Severe suicidal depression as a response to financial stress and family responsibilities was also recognised by a 47-year-old Village Health Worker and leader in the community. He claims to have seen a rise in suicide as a result of poverty-related stress and depression. He also highlights a reluctance to share problems, which, for him, can have a damaging impact on the brain:

‘the bite in poverty…people are struggling to take care of their family but want to keep their problems to themselves. When you think to yourself it becomes too much for your brain… People get ideas that they’re useless to their family, who would be better off without them.’ (Participant 2, 47 year old man, Village Health Worker)

Stress, depression and anxiety are frequently (*n* = 8) described as ‘overthinking’ or ‘thinking too much’ in response to economic adversity. Here, ‘overthinking’ is associated with the idea that thoughts are uncontrolled, intrusive and thus damaging to the brain:

‘If you think a lot your heart beats at a high speed to the point that if they bring bad news suddenly you can just faint…it can go to your brain, then it can disturb your brain.’ (Participant 3, 60 year old, woman)

‘too much thoughts, when you think a lot your brain will get exhausted, that’s how you get mad.’ (Participant 4, 44 year old, woman)

Three people identified stress as an issue for women in particular, who have responsibilities for their family, such as this 39-year-old single mother. She recognises her drinking as a response to the responsibilities she ‘cannot handle’:

‘Women have a lot of stress and responsibility and nowhere to start… I was passing through a lot of things…Like I had so much stress and sometimes I fainted…When I think a lot, I also sometimes end up drinking and vomiting…I have responsibility that I cannot handle.’ (Participant 5, 39 year old, woman)

Some respondents expressed sympathy and understanding for people who resort to drinking alcohol or taking drugs, associating it with high unemployment and financial stress. Others are critical of drug and alcohol consumption and the impact it’s having on family and community life:

‘Their head is now big; they don’t understand things… They can’t dig, they’re leaving home for nothing…it’s spoiling home.’ (Participant 6, 51 year old, woman)

Several interviewees (*n* = 14) believe that mental health problems are because of spiritual causes, including ancestral curses (*n* = 1), spiritual punishments for crimes such as murder (*n* = 2), attacks from ‘bad spirits’ (*n* = 2) and ‘witchcraft’ (*n* = 9), intentional psychological harm inflicted by a curse from relatives or neighbours. On hearing our initial general question about mental health, five people in separate interviews immediately responded as follows:

‘first njagga [*drugs*], second witchcraft.’ (Participant 7, 58 year old, woman)

Four people had direct experiences with relatives thought to have been bewitched, such as one interviewee whose nephew was cursed by a cousin and healed by a ‘witch doctor’. Two people said they had never witnessed witchcraft causing mental illness themselves but had ‘heard rumours’. These rumours are supported by the onset and presentation of mental health problems, such as seemingly sudden attacks, severe or violent manifestations of psychological problems.

## Care-seeking practices

Despite the relatively common association between mental health problems and supernatural causes (*n* = 14), and the perceived inaccessibility of medical treatment, fewer respondents (*n* = 7), including three health workers, referred to treating mental health problems with traditional or spiritual healing methods and only one person had a personal experience of a relative being treated by a traditional healer. The relative infrequency of traditional or spiritual care-seeking was sometimes (*n* = 4) contrasted with rural areas because of hospital services being more accessible in the city.

Many participants (*n* = 15) said they are only aware of available treatment for mental health problems at the national psychiatric hospital, Butabika. This includes two of the local private clinicians interviewed, who said that in the case of enquiries regarding mental health problems, they immediately refer people to Butabika. This is despite the availability of psychiatric services at the regional government hospital within walking distance. The idea that Butabika is the only option for treatment might delay help-seeking, as Butabika is considered a last resort once problems have become severe. As observed by a Village Health Worker and local leader:

‘They leave it too late before going to hospital… Butabika is the only place people know, there’s nowhere in-between to go.’ (Participant 2, 47 year old man, Village Health Worker)

The perceived costs involved in hospital treatment also prevent help seeking, as this 50-year-old man pointed out:

‘If your family has a lot of money and you run mad, they bring you to Butabika. If not, they just leave you.’ (Participant 8, 50 year old, man)

With the perceived inaccessibility of professional mental healthcare, seeking advice and counselling from friends and relatives is recognised by respondents as a preferable solution to mental health problems such as stress and ‘over thinking’. Some participants (*n* = 7) said they would prefer to share their worries with someone they know and trust in their community, someone who can relate to them and therefore give more actionable advice:

‘If I notice someone is stressed or depressed, I befriend them, after all that person needs someone to talk to like a counsellor or friend… A Doctor can’t finish such a problem, it’s a friend who can help you.’ (Participant 8, 50 year old, man)

Some of the women interviewed said that they have people coming to them for advice and counselling. Often, they said they would advise someone with stress to ‘stop thinking’, as in these examples:

‘If someone is stressed, it’s hard to tell unless they tell you and some people don’t talk… If they did, I would advise them to stop over thinking as it’s not good for you.’ (Participant 4, 44 year old, woman)

‘I always advise him [her son who has depression] to stop being stressed…I think it’s the only advice those doctors’ could also give him.’ (Participant 9, 45 year old, woman)

‘When people are stressed, I tell them don’t think of that thing, it’s you who wants to think of it.’ (Participant 10, 80 year old, woman)

Whilst seeking advice from someone within the community is sometimes the preferred approach, others (*n* = 5) observed that there can be a reluctance to discuss private mental health concerns. As a 40-year-old single mother, who herself had been struggling with depression and alcohol use, put it:

‘People fear talking about it a lot.’ (Participant 11, 40 year old, woman)

Two local leaders also report that they offer free counselling to others within the community. One of them, a local elected councillor for women, received training from an non-governmental organisation (NGO) to provide empathetic and confidential counselling for other women with depression. Having had severe depression herself as a result of financial stress, she had found it hard to talk about her problems with others in the community:

‘You may have problems then you go and tell somebody, she may start spreading it…They will not help you, others will even laugh at you.’ (Participant 1, 49 year old woman, Women’s Leader)

She plans to form a group for widows to support their mental health and their finances. The second, a 60-year-old man, provides counselling for mental and spiritual sickness:

‘I’m one of the people who counsels people…I normally deal with people who have issues at home, people who are sick, mentally sick, spiritually sick…I deal with people, pray for somebody’s soul, read the bible and you find somebody getting delivered, somebody getting okay or something like that… they say go to mzee (old man)…it is very free, I don’t want even to be paid.’ (Participant 12, 60 year old man, Religious Leader)

He believes that, like Doctors, he can encourage people who are suffering from stress and emotional or spiritual problems to rest. Having converted to Pentecostal Christianity or becoming ‘born again’, in 2004 and recovering from alcoholism, he also leads people to the Church. Five other respondents also observed that they had recovered from alcohol addiction after becoming born again, advocating that as a solution for others:

‘Ever since I got born again, I stopped drinking… the Church has helped me a lot, counselling and prayer, I changed.’ (Participant 12, 60 year old man, Religious Leader)

‘If I couldn’t convene with God I would be in very bad shape, so prayer helps me a lot. I think others should pray as it stops a lot of people from getting sick.’ (Participant 13, 65 year old, woman)

As in these citations, some women have explained how they use prayer as a daily ritual to manage their own stress, as a source of strength and comfort and a way to acknowledge and share their worries.

Some participants (*n* = 5), including three health workers, also advocate for further health education within the community, so that people with mental health problems and their caretakers can gain more information. One older man discussed his 28-year-old nephew who has become addicted to ‘mirunji’ (herbal stimulant); the family find that they do not receive any advice in the health clinics they take him to, other than being told to stop taking the drugs. They tried to persuade him to become born again and took him back to the village, but he returned to Kampala. His Uncle said they would now appreciate being able to access more advice. One 45-year-old participant also advocated for health education in order to overcome mental health stigma. Having observed violence towards a young man with mental health problems, she said:

‘It’s not good to beat such a person, because it’s their mind it’s not them. Sometimes people just do it even though they know he’s not ok…people need to be taught in the community, as sometimes you don’t know what spoils people’s heads.’ (Participant 14, 45 year old woman)

## Discussion

The findings show that common mental health disorders, such as addiction, depression and anxiety - which can be referred to as ‘overthinking’ or ‘thinking too much’ - are often primarily handled within communities, with the help available from various sources including family, friends and religious counsellors. Some research participants felt that they would be unwilling to share personal problems with a doctor, who they saw as less able to advise them in place of someone who knows them and would therefore be able to give more relevant and accessible advice. Faith-based personal strategies of managing stress, including prayer and born-again Christianity, are also often advocated.

These non-medical approaches are not factored into what WHO have identified as the ‘treatment gap’ for ‘moderate and mild’ mental disorders, estimated to affect 1.67 million people in Uganda.^6^ Our study suggests that whilst mental health problems are considered to be prevalent, there are deterrents to seeking medical or therapeutic help, as mental health services such as those in the national psychiatric hospital Butabika are stigmatised or considered to be inaccessible or expensive. With the scarcity and high cost of mental health services in Uganda, sometimes ‘overestimated’^10^, there is a challenge to promote awareness of treatment options and to make treatment more acceptable and accessible.^27^ There can also be deterrents to seeking advice about personal, mental and spiritual problems from familiar people, with some people expressing fears of judgement and preferring to manage problems themselves.

The interview responses show that in all discussed explanatory models of mental health problems, in relation to financial stress, interpersonal conflict, drugs and spiritual issues, psychological problems are understood to be socially determined and managed. Mental health problems are most commonly attributed to financial stressors such as unemployment, disappointment, poverty and the pressure of providing for children and paying school fees, which some note as a particular stressor for women. This shows how mental health problems can themselves be considered a form and consequence of social suffering, as well as a psychiatric disorder.^18^

The use of drugs such as marijuana and alcohol are widely considered to be a significant cause and consequence of mental health and social problems in the community. Mental health problems are also frequently discussed by respondents in terms of their social outcomes, such as disruption to family and community life. This is in line with Okello and Neema’s study of explanatory models of depression in Uganda, which found that only when mental health problems become ‘socially disruptive’,^24^ treatment is sought. Okello and Neema also found that ‘psychotic symptoms’ are likely to be understood as a result of spiritual causes such as ‘poor relations between the living and the dead’,^24^ whereas ‘non-psychotic symptoms’ of mental health problems are conceived as an ‘illness of thoughts’ related to psychosocial problems such as poverty.^24^ This is reflected in our study, which found nonpsychotic symptoms of anxiety, depression and addiction are often attributed to socioeconomic problems. This can be expressed as ‘over-thinking’, thoughts that are uncontrolled and damaging to the brain, said to be a common idiom of distress or unhappiness around the world,^28^ alongside increasingly globalised diagnostic labels of depression^29^ or ‘post-traumatic stress’.^30^ The idea of damage to the brain caused by ‘over-thinking’ is also evident in citations from women who advise people, particularly their (grand) children to ‘stop thinking’; this advice also implies an expectation that thoughts are controllable, and that ‘thought disorders’ demonstrate an inability to personally manage social issues.

Like other studies in Uganda, this research has shown that misfortune or sickness can often be attributed to ‘interpersonal conflict’,^31^ manifest in spiritual punishment from ancestors, relatives co-wives or neighbours. Spiritual explanations reflect ‘phenomena that some people wonder about’,^30^ rather than universal or uncontested beliefs. In this study, ‘witchcraft’ is commonly attributed to mental health disorders amongst interview participants yet reporting of spiritual treatment seeking was relatively rare. In contrast, other studies in Uganda have shown that mental health problems are the most common problem traditional healers treat,^32^ and that people seek traditional healing before, during and after medical treatment.^10^ It is possible that this is because of a distinction between urban and rural help-seeking practices, with people living in Kampala more likely to first seek help at the hospital because of greater proximity and availability in the capital city. Self-reporting respondents may also prefer to keep experiences of traditional or spiritual healing private. It may also be because of born-again Christianity.^24,33^

### Limitations

The qualitative study had limitations, as respondents were made up of a convenience sample and therefore are not necessarily generalisable to the entire community. Further research could use a larger, randomised sample of respondents, which could also benefit from a mixed method approach. The research was conducted primarily by a British researcher, which presents limitations in relation to interpreting meaning associated with mental health. This limitation has been considered in the long-term, open and grounded approach, to ensure familiarity with participants and relevant terminologies. The research also relied on close collaboration with a team of Ugandan health practitioners and co-researchers, who were involved throughout the research process, from study design, data analysis to dissemination.

### Strengths

The strength of our study lies primarily in the ethnographic approach to health research, suitable for understanding the complexity of mental health perceptions and care-seeking preferences. The sample of 56 participants allowed for a broad and ‘richly textured understanding’ of mental health experiences, perceptions and attribution, without compromising depth.^34^ The research accommodates a breadth of responses from participants who might not typically engage with online or hospital-based research on mental health. This has shed light on a more socially situated understanding of mental health and its’ management in Kampala. The study is also strengthened by the perspectives of various (mental) health workers in Kampala, including participating authors, who validated the methodology, analysis and manuscript.

## Conclusion

The findings from our study come from a diverse sample of people over 37 years old and health workers. This offers a broad understanding of mental health attribution, experience and treatment-seeking preferences. Prevalent attributions of mental health disorders include drugs and alcohol, economic problems, interpersonal and spiritual issues. These findings implicate a ‘socially situated’ understanding of mental health in Uganda.^32^ This stresses the need to understand the ways social perceptions are influential in determining mental health disorders and their treatment,^35^ for example, stigma as a deterrent to seeking help in hospitals, or even to seeking advice from neighbours or relatives. Anticipated stigma and costs evidently present a barrier to visiting the available mental health services. As other studies in Uganda have shown, the inaccessibility of services may be overestimated, for example, none of the participants in this research referred to available mental health resources at the nearby government hospital. Instead, some people said that they prefer to manage their own mental health problems or to seek support from known people within their community rather than medical doctors. Faith-based strategies such as prayer have evidently played a key role for some participants in overcoming common mental health problems such as stress, anxiety, depression and addiction. Overall, our study advocates for formative research to understand existing mechanisms of coping in the community, integrating their preferences into treatment as appropriate and paying attention to systematic issues that affect mental health.

### Recommendations

These findings support recommendations to provide easily accessible, low cost and discrete professional counselling services. Private or confidential counselling options could provide a helpful addition to existing home-based and community-based methods. Efforts can also be made to integrate services at community level, for example, through community-based groups, services accessible from home and hospital outreach. Raising awareness of available services at regional hospitals, if sufficiently resourced, can improve their up-take and prevent people from delaying treatment until presenting in an acute phase at the national hospital. In particular, outreach could target people who are influential in their communities and often already providing mental health advice, such as local politicians, religious leaders and community elders. Services can also provide mental health support by incorporating financial assistance, in line with other successful initiatives aimed at reducing depression in Uganda.^36^
